# The Effect of Competition Between Two Swan Species: Nesting Site Selection and Reproductive Success

**DOI:** 10.3390/ani16060901

**Published:** 2026-03-13

**Authors:** Wojciech Szewczuk, Iga Słomkiewicz-Szewczuk, Zbigniew Kasprzykowski

**Affiliations:** 1 Faculty of Science, University of Siedlce, Prusa 14, 08-110 Siedlce, Poland; zbigniew.kasprzykowski@uph.edu.pl; 2 Independent Researcher, 01-708 Warsaw, Poland

**Keywords:** interspecific competition, range expansion, habitat selection, climate change, Whooper Swans, Mute Swan

## Abstract

Climate change-driven range expansions are creating novel interspecific interactions that may significantly impact the breeding success of established resident species. This study examines the ecological consequences of competition between Mute Swans (*Cygnus olor*) and expanding Whooper Swans (*Cygnus cygnus*) in central and eastern Poland. We monitored 80 Mute Swan breeding pairs across fishpond complexes using UAV-based surveys to assess habitat selection and reproductive output in sites with and without Whooper Swan presence. Mute Swans breeding alongside Whooper Swans selected nest sites deeper within reed vegetation and showed altered habitat preferences compared to pairs breeding without competition from a related species. Significantly, reproductive output was reduced in competitive environments, with pairs breeding in areas without Whooper Swans producing considerably more offspring than those coexisting with the expanding species. These results demonstrate that the expansion of a given species’ range can cause immediate costs to local species, both via direct confrontations and through the impact on the quality of the occupied habitat. The substantial reduction in breeding success suggests that interspecific competition may have population-level consequences for established waterbird communities.

## Simple Summary

This study investigates the competitive interactions between the expanding Whooper Swans (*Cygnus cygnus*) and the resident population of Mute Swans (*Cygnus olor*) for nesting sites in fishponds. Monitoring 80 Mute Swan pairs via UAV surveys revealed that swans breeding alongside Whooper Swans selected deeper reed sites and showed significantly reduced reproductive success. These findings highlight the immediate ecological costs associated with the spread of expanding species, providing useful information for habitat management in the context of climate change.

## 1. Introduction

Global environmental change is reshaping avian communities through various alterations in species distributions, with breeding ranges of numerous bird species experiencing rapid shifts [1]. These uneven shifts create new overlaps and interactions among species that did not previously coexist [2]. Some distributional changes extend beyond simple geographical relocations. They alter animal populations by creating novel relationships and competitive interactions [3]. Recent research demonstrates that many Northern Hemisphere bird species are shifting their distributions northward, with changes in latitudinal optima averaging about 1.5 km per year [2].

In bird communities, interspecific territorial behaviour has become a key mechanism, where territorial aggression between species is strongly linked to reproduction and resource overlap during breeding seasons [4]. Competitive interactions are particularly pronounced when morphologically similar species with overlapping ecological niches meet in breeding habitats. This often results in asymmetric outcomes that favour behaviourally dominant competitors [5,6]. Aggressive species undergoing range expansion can alter the reproductive strategies and success of established residents. Subordinate species may adopt alternative nesting behaviours or experience reduced breeding output [7]. Moreover, studies have shown that the mere presence of waterbirds can reshape local habitat conditions by intensifying grazing pressure on submerged vegetation and altering nutrient dynamics, thereby contributing to broader ecosystem-level changes that may further disadvantage co-occurring species [8,9,10].

Waterbirds represent particularly valuable indicators of environmental change, as their rapid demographic responses to climatic variation provide insight into broader ecosystem transformations [11]. A recent study has revealed considerable variation in the responses of different species of waterbirds to changing environmental conditions, with some populations showing remarkable resilience while others face serious declines [12]. Breeding success, habitat selection, and population dynamics are strongly influenced by density-dependent processes and interspecific competition. This is particularly evident when expanding populations encounter established residents in newly colonised areas [13].

The Whooper Swan *Cygnus cygnus* showed dynamic range expansion facilitated by changing climatic conditions [14]. Censuses of Icelandic Whooper Swans indicate a continuing south-eastern shift in wintering distribution, likely driven by milder winters and changing environmental conditions, which may alter habitat use [15,16]. Population monitoring across Northern Europe indicates sustained growth rates exceeding 10% annually in Poland. The species has established new breeding populations far beyond its historical range [17]. This expansion has been accompanied by increasing utilisation of anthropogenic habitats, particularly fish farming facilities, which provide stable water conditions and enhanced food resources [18].

The expansion of Whooper Swans increases territorial behaviour and competition with other native waterfowl [19]. Behavioral studies show that Whooper Swans often act aggressively towards other water bird species and usually dominate in competition for resources [20]. Recent demographic analyses suggest that nature reserves and protected areas play crucial roles in supporting expanding Whooper Swan populations, with survival rates in managed areas significantly exceeding those in unprotected habitats [21]. Unlike the Whooper Swan, Mute Swan *Cygnus olor* populations have remained relatively stable in many regions in recent years [22,23]. However, breeding swans may experience reduced foraging efficiency, altered time–activity budgets, or increased physiological stress when confronted with new territorial competitors [24,25].

Central and Eastern Europe represents an excellent region in which to study such interactions, as both swan species have begun to encounter each other here in recent decades, and competition between them is highly dynamic [18]. The aim of this study is to test two interrelated hypotheses. The first hypothesis (H_1_) assumes that there are differences in habitat parameters between Mute Swans nesting in the same habitats as Whooper Swans and Mute Swans breeding without Whooper Swan neighbours. We expect that Mute Swans nesting together with Whooper Swans would place their nests in more concealed locations than Mute Swans breeding separately. In habitats without interspecific competition, Mute Swans choose locations close to larger open water, providing sufficient take-off space, and a preference for heavily emergent vegetation has not been observed [26,27]. Human pressure has also not been observed to influence this species’ tendency to conceal its nests [18]. Therefore, apart from moving to less attractive nesting sites, nesting deeper in the reeds may represent another way for Mute Swans to avoid interspecific competition. The second hypothesis (H_2_) is that reproductive success of Mute Swans, defined as the number of cygnets, is lower when competing for breeding territories with Whooper Swans. Testing these two hypotheses may help describe how native species respond to expanding species. The relatively recent nature of this sympatric relationship means that long-term evolutionary adaptations to competition are unlikely, making the reproductive responses very valuable for understanding the immediate consequences of range expansion-driven species interactions.

## 2. Materials and Methods

### 2.1. Study Area

**Figure 1:**
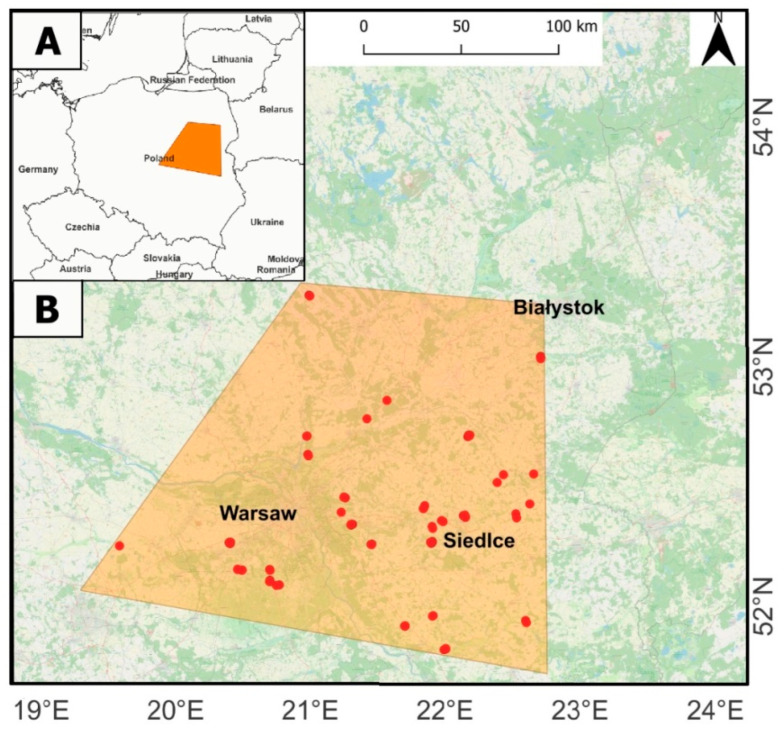
Map of the study area (**A**) and location of fishpond with Mute Swan nesting sites ((**B**) red dots). Created by research team using QGIS version 3.34.13.

The survey was conducted in central and eastern Poland (52.369703, 21.552751), covering an area of approximately 33,000 km^2^ across fishpond complexes distributed throughout the provinces of Mazovia and Podlasie (Figure 1). These artificial wetlands primarily serve fish-farming purposes, predominantly breeding carp (*Cyprius carpio*), with water levels seasonally managed in connection with spawning activities and vegetation maintenance. The fishponds are characterised by variable vegetation coverage, with the most common species being reed (*Phragmites australis*), reedmace (*Typha* sp.) and sedge (*Carex* sp.). This habitat type has been identified as a preferred nesting habitat for the expanding Whooper Swan population within the study area, whilst also supporting established Mute Swan breeding habitats over recent decades [18].

We focused exclusively on fishpond habitats to examine breeding interactions where interspecific competition is strongest. The selection was based on earlier findings indicating that Whooper Swans predominantly occupy fishpond complexes, whilst Mute Swans are more evenly distributed between habitat types [28]. Each pond complex was treated as a single biotope. Although both species may use different ponds within the same complex, they are aware of the presence of their neighbours. This is due to the strong territorial behaviour of Whooper Swans, which often fly around the entire area of suitable habitat and interact with other individuals of both species [24].

### 2.2. Field Procedures

Field surveys were conducted from 1 April to 14 July 2023 and from 7 April to 12 July 2025, incorporating two distinct phases of data collection. Observations from different years were treated as independent because fishpond habitats in the study area undergo substantial interannual changes due to fish-farming practices. Water levels are frequently modified, reedbeds are removed or regenerated, and individual pond basins may be drained or flooded, leading to marked differences in nesting conditions between seasons. Consequently, the same location in different years does not represent repeated measurements of a stable territory. The methodology is based on proven measurement techniques using unmanned aerial vehicles (UAVs), which have previously been approved for monitoring swans [29], whilst adapting methods specifically for cygnet number assessment in competitive breeding environments. The first phase commenced in early May with comprehensive surveys to identify active breeding pairs of both swan species. We followed protocols established in a previous study and used UAVs to locate nests and identify breeding pairs [18]. Two drone models were employed: the DJI Mavic Mini and the DJI Mavic Enterprise Thermal. UAV flights were conducted at altitudes of 50–80 m AGL (Above Ground Level), which is an appropriate flight level to avoid any disturbance whilst maintaining sufficient image resolution for species identification [30]. We conducted systematic transect surveys across each fishpond complex, paying particular attention to areas with emergent vegetation where breeding swans were most likely to occur. All active nests were georeferenced using geotagged photographs taken directly above nesting platforms. Species identification was confirmed through detailed examination of adult birds, particularly on the basis of morphological criteria including bill coloration and neck posture. The second phase involved systematic counts of cygnets during June and July, when most cygnets had hatched but remained in close proximity to adults. We selected this timing to capture early reproductive success, when cygnets remained easily recognisable and countable. Each breeding territory was checked several times during the whole breeding season until adult birds were observed accompanied by chicks at the appropriate stage of growth. Due to the extended breeding season, this required 4 to 7 visits. Data was collected on 78 broods, of which 65.4% were successful (Table S1, Supplementary Material). Chicks were counted between hatching and reaching 1/4 of the body size of an adult. Drone surveys were repeated at each previously identified nest location, with flights conducted during optimal conditions (clear weather, minimal wind, and good visibility) at the appropriate flight height (minimum 50 m AGL). All visible cygnets were recorded for each family group. Efforts were made to avoid double-counting by surveying each pond complex in a single session and maintaining detailed records of family group locations. Particular attention was paid to distinguishing between fishponds supporting single-species breeding (either Mute Swan or Whooper Swan pairs only) versus mixed-species sites where both species nested within the same pond complex. This categorisation enabled us to compare reproductive success under different competitive scenarios. By maintaining consistent survey timing, standardised cygnet identification criteria, the data collected enabled meaningful comparisons between single-species and mixed-species breeding environments. Each occupied nesting territory was checked several times until the loss at the incubation stage was confirmed or the early survival of cygnet (first 3–4 weeks post-hatching) was determined. This category of nestling growth could provide information on the most vulnerable period of swan reproduction, when parental care is most intensive and ecological conditions are critical for offspring survival. The parameter determining the variation in the timing of breeding was the date on which chicks aged 3–4 weeks were observed. The dates were scaled such that day 1 was the day when the first adult birds leaded chicks at this stage of growth was found and day 48 was the day the last date was noted.

### 2.3. Spatial Data Analysis

Two sets of geotagged photographs—one showing swan nest locations and another showing Mute Swan family groups—were imported into QGIS software version 3.34.13. On the basis of spatial relations, for each nest, the additional data were appended (Table 1).

Five macro-habitat variables surrounding each swan’s nest were selected. Distance to the reeds edge (ReedDist), distance to the dyke (DykeDist) and rate of reed (ReedRate) best reflect nest concealment. In turn, distance to nearest forest (ForestDist) and distance to nearest built-up area (BuiltDist) differentiate the choice of nesting sites for both competing swan species [18]. All measurements were taken on the basis of Landsat 8 images (Google Earth) and verified with Sentinel-2 satellite imagery taken in May 2023 and 2025 (Table 1).

### 2.4. Statistical Analysis

All statistical analyses were performed in R (version 4.5.1) [31]. A general linear model (GLM) with logit link function and binomial error variance was used to compare the species’ macro-habitat preferences. The dependent variable was the occurrence category of Mute Swan (binomial variable: 0 = separate, 1 = competitive). The global model was developed according to the following formula: Category ~ ReedDist + ReedRate + DykeDist + BuiltDist + ForestDist. Prior to model selection, multicollinearity among predictors was checked using the Variance Inflation Factor (VIF). All VIF values were below the threshold of 2, indicating no problematic multicollinearity [32].

Model selection was conducted using the information-theoretic approach (AIC) [33]. A set of all possible models was generated from the global model using “dredge” function from the MuMIn package (version 1.47.1) [34]. Models were ranked according to their AICc values, with lower values indicating better model fit. Model fit was assessed using McFadden’s pseudo R^2^ method [35]. Residual diagnostics were conducted using the DHARMa package (version 0.4.7) to test for dispersion and zero-inflation issues. Model predictions were extracted using the ggeffects package (version 2.3.0) [36], and graphical representations were produced with the ggplot2 package (version 4.0.2). To examine differences in the date when chicks aged 3–4 weeks were identified and in the number of cygnets between competitive (COM) and separate (SEP) types of Mute Swan nests, a Mann–Whitney U test was applied due to the non-normal distribution of count data. Descriptive statistics were calculated for each group, and effect size was assessed through comparison of group medians.

## 3. Results

**Figure 2:**
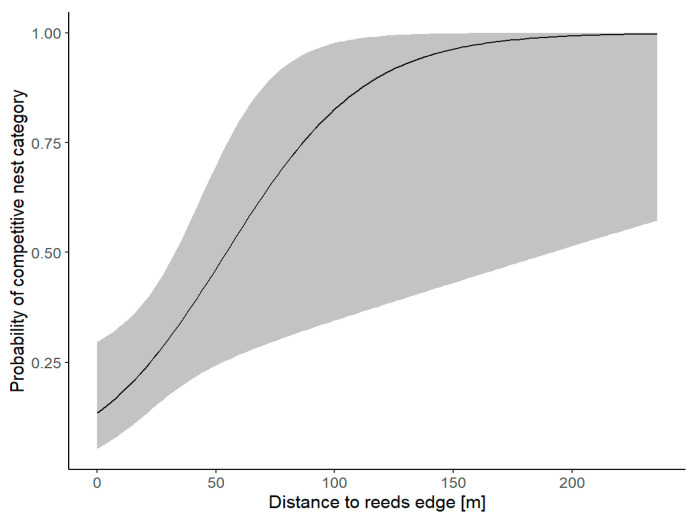
Predicted probability of competitive nest category vs. distance to reed edge.

The environmental model demonstrated adequate fit to the data (McFadden’s pseudo R^2^ = 0.394, AIC = 77.3, N = 80). On the basis of the AICc criterion, the full set of possible predictors was included in the final model (Table 2). Distance to reed edge emerged as the only significant predictor of nest site selection of Mute Swan competing with Whooper Swan (Table 3). The probability of competitive nesting sites of Mute Swan increased with distance to the age of vegetation patches (Figure 2).

Distance to dyke and distance to built-up area were close to statistical significance, whilst reeds percentage and distance to forest were non-significant predictors (Table 3).

**Figure 3:**
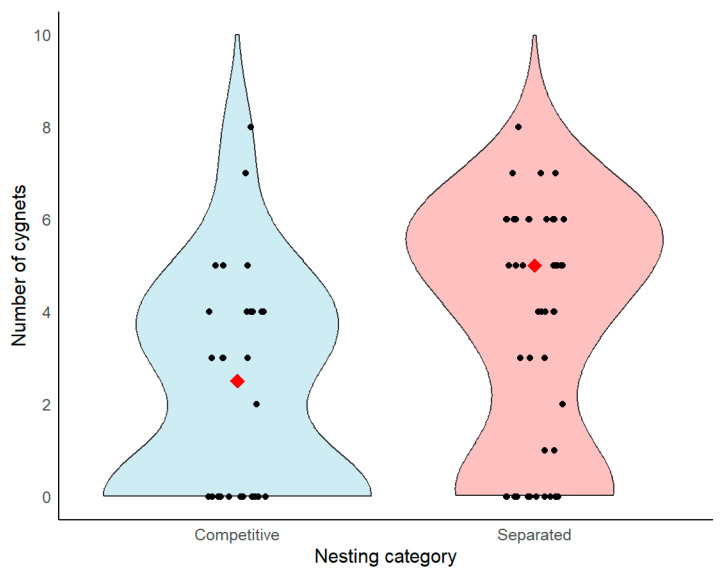
The violin plot displays raw cygnet counts by nesting category, with black points representing individual observations and red diamonds representing medians for each category.

No significant differences between separate and competitive types of Mute Swan nests were found in the date of identified chicks aged 3–4 weeks accompanied by adult birds (Mann–Whitney U test, Z = 0.34, *p* = 0.732, N = 53). However, separate nest types had higher reproductive success (median = 5.0 cygnets, IQR = 0.75–6.0, N = 46) compared to competitive ones (median = 2.5 cygnets, IQR = 0.0–4.0, N = 32, Figure 3), indicating reduced reproductive output under interspecific competition. The difference in the number of Mute Swan cygnets between nest types was significant (Mann–Whitney U test, Z = 2.79, *p* = 0.004, N = 78).

## 4. Discussion

Our study indicates that Mute Swans react to the appearance of a new competitor by placing their nests deeper in the reeds. This is evidenced by the greater distance from the edge of the reeds to the nests in cases where both species coexist. Earlier investigations demonstrated that Mute Swans modify their nesting strategies by selecting more concealed and isolated locations when breeding in proximity to Whooper Swans, representing an apparent adaptive response to competitive pressure [28]. These findings suggest that this behavioural response may be insufficient to mitigate reproductive costs associated with interspecific competition. Competition itself may also entail costs. Moreover, previous habitat-focused research revealed distinct habitat preferences between the two swan species, with Whooper Swans favouring fishponds surrounded by forests whilst Mute Swans selected more open areas near human settlements [18]. When sharing the common pond complex and concealing their nests in highly vegetated niches, Mute Swans may venture beyond their optimal habitat. Whilst selecting more concealed nesting sites may reduce direct confrontations, these locations may simultaneously compromise other breeding conditions.

The investigation demonstrates a negative impact of the presence of nesting Whooper Swans on the reproduction of Mute Swans, with pairs breeding without interspecific competitors producing significantly more cygnets than those coexisting with the expanding Whooper Swan populations. This suggests that breeding Mute Swans experience costs when sharing pond complexes with territorial Whooper Swans. Other research on avian breeding success has increasingly recognised that competitive environments can substantially reduce reproductive output even without direct physical displacement, through reduced access to optimal foraging areas or imposition of energetic costs associated with increased vigilance and territory maintenance [4]. This interpretation aligns with suggestions that behavioural plasticity in competitive environments often involves fitness trade-offs rather than cost-free solutions. Differences in reproductive success between nesting categories occurred alongside significant environmental differences. This makes it difficult to distinguish direct competitive effects from habitat-mediated effects. Pairs nesting together with Whooper Swans in ponds have a greater reeds rate, placing their nests deeper in reeds patches, which may represent either: (1) direct avoidance behaviour in response to Whooper Swan presence; (2) the influence of habitat factors that independently affect both species distributions and reproductive success of Mute Swan.

While our results demonstrate clear reproductive consequences of interspecific competition, the limited sample size, caused by the relatively small number of places where both swan species nest together, prevents unequivocal attribution of the most important causes of the observed effects. Nevertheless, ecological processes are complex. The impact of competition should therefore be considered holistically, as it may involve multiple components of varying intensity [26,37]. The substantial reproductive costs documented in this study align with the assumption that interspecific competition should generate strong pressure for spatial or temporal separation between competing species [4]. Analyses of swan population dynamics show that adult survival has a greater impact on population size than juvenile apparent survival [13]. Given that swan populations exhibit relatively slow demographic responses due to extended pre-reproductive phases and high adult survival rates, sustained reproductive interference could produce delayed but pronounced population declines that manifest years after initial competitive pressure.

## 5. Conclusions

The combination of behavioural modifications and reproductive costs suggests that Mute Swan populations face various challenges that may give rise to different scenarios. Future research priorities should include longitudinal monitoring of population demographics to assess whether observed reproductive interference translates into population-level declines and investigation of potential adaptive responses in breeding behaviour or habitat selection.

## Figures and Tables

**Table 1:** Factors used to explain differences in two categories of Mute Swan nests.

Code	Description
ReedDist	The distance between the Mute Swan’s nest and the edge of the reeds within which the nest was concealed (km)
ReedRate	Proportion of reeds area to open water area within a single water body
DykeDist	The distance from a Mute Swan’s nest to the nearest dyke within particular water body (km)
ForestDist	The distance to the nearest forest with an area of at least 1 ha (km)
BuiltDist	The distance to the nearest built-up area (km)

**Table 2:** Top predictor sets ranked by AICc criteria. Degree of freedom (df), model log-likelihood (LL), corrected AIC (AICc), difference between the model and the best model in the data set (ΔAIC), and weight for the model (AICw).

Model	df	LL	AICc	ΔAIC	AICw
Intercept + ReedDist + ReedRate + DykeDist + BuildDist + ForestDist	6	−32.650	78.450	0.000	0.150
Intercept + ReedDist + DykeDist + BuildDist + ForestDist	5	−33.828	78.466	0.017	0.149
Intercept + ReedDistp + ReedRate + DykeDist + BuildDist	5	−33.862	78.534	0.084	0.144
Intercept + ReedDist + ReedRate + BuildDist + ForestDist	5	−34.244	79.299	0.849	0.098
Intercept + ReedDist + ReedRate + DykeDist	4	−35.389	79.312	0.862	0.097
Intercept + ReedDist + DykeDist + BuildDist	4	−35.481	79.496	1.046	0.089
Intercept + ReedDist + BuildDist + ForestDist	4	−35.505	79.544	1.094	0.087
Intercept + ReedDist + ReedRate + DykeDist + ForestDist	5	−34.588	79.987	1.537	0.069
Intercept + ReedDist + ReedRate + BuildDist	4	−35.754	80.042	1.592	0.068

**Table 3:** Results of best model comparing habitat factors between nesting categories.

Factor	Estimate	Standard Error	z-Value	*p*-Value
(Intercept)	−3.815	1.015	−3.758	<0.001
ReedDep	0.034	0.014	2.405	0.016
ReedRate	0.021	0.014	1.514	0.130
DykeDist	0.016	0.009	1.742	0.082
BuildDist	0.002	0.001	1.842	0.065
ForestDist	−0.002	0.002	−1.383	0.167

## Data Availability

Datasets supporting the reported results can be found at the Mendeley Data Repository: https://data.mendeley.com/datasets/bfj4p5nc33, accessed on 5 December 2025.

## Supplementary Materials

The following supporting information can be downloaded at: https://www.mdpi.com/article/10.3390/ani16060901/s1. Table S1: Number of nests in the categories of Mute Swan cygnet number in competing and separate types of nests.
