# Supplementary material for: The Effect of Competition Between Two Swan Species: Nesting Site Selection and Reproductive Success
Source: Animals (Basel). 2026 Mar 13;16(6):901. doi: 10.3390/ani16060901 (PMC13023284; doi:10.3390/ani16060901)
Supplement: Supplementary file 1 [file animals-16-00901-s001.zip › animals-4115162-supplementary.pdf]

Supplementary Appendix for the article:

The effect of competition between two swan species: nesting site selection and reproductive success, Szewczuk et al.

Table S1: Number of nests in the categories of Mute Swan cygnet number in competing and separate types of nests.

Number of cygnets	Number of nests in competitive category	Number of nests in separate category	Total
0	15	12	27
1	0	1	1
2	1	1	2
3	5	3	8
4	6	5	11
5	3	8	11
6	0	12	12
7	1	3	4
8	1	1	2
